# Relative sensitivity of anterior nares and nasopharyngeal swabs for initial detection of SARS-CoV-2 in ambulatory patients: Rapid review and meta-analysis

**DOI:** 10.1371/journal.pone.0254559

**Published:** 2021-07-20

**Authors:** Yaolin Zhou, Timothy J. O’Leary

**Affiliations:** 1 Department of Pathology & Laboratory Medicine, Brody School of Medicine, East Carolina University, Greenville, North Carolina, United States of America; 2 Office of Research and Development, Veterans Health Administration, Department of Veterans Affairs, Washington, District of Columbia, United States of America; 3 Department of Pathology, University of Maryland School of Medicine, Baltimore, Maryland, United States of America; Universidad Nacional de la Plata, ARGENTINA

## Abstract

Nasopharyngeal (NP) swabs are considered “gold standard” for diagnosing SARS-CoV-2 infections, but anterior nares or mid-turbinate swabs (nasal swabs) are often used. We performed a meta-analysis comparing the sensitivity of nasal and nasopharyngeal swabs against a composite reference standard for the initial diagnosis of SARS-CoV-2 infection in ambulatory patients. The study is registered on PROSPERO (CRD42020221827). Data sources included studies appearing between January 1, 2020 and March 20, 2021, identified by searches of PubMed, medRxiv and bioRxiv. Studies included at least 20 subjects who simultaneously provided nasal and nasopharyngeal specimens for reverse transcription-polymerase chain reaction testing, and for which confusion matrices could be constructed. Authors individually assessed studies for inclusion and compared assessments. Each author independently extracted all data elements; differences were reconciled by review of initial data sources. Extracted data included specimen site, patient characteristics, collection site, and confusion matrices comparing results for nasal and nasopharyngeal swabs. Assessed against a composite reference standard, anterior nares swabs are less sensitive (82% - 88%) than nasopharyngeal swabs (98%). For populations with 10% specimen positivity, the negative predictive values of all swab types were greater than 98%. Mid-turbinate and anterior nares swabs seem to perform similarly. The lower sensitivity associated with nasal swab SARS-CoV-2 diagnosis is justified by the ability to screen more patients and reduced personal protective equipment requirements. Our conclusions are limited by the small number of studies and the significant heterogeneity of study designs and study outcomes.

## Introduction

Rapid identification of SARS-CoV-2 viral infection is important for patient treatment, isolation of asymptomatic carriers, and following the community spread of infection [1]. Although flocked-swab nasopharyngeal samples (NPS) have constituted a “gold standard” upper respiratory system specimens, samples obtained by swabbing the anterior nares (ANS, also known as “nasal”) are now often employed, because these samples can be self-collected, reducing both exposure and the need for personal protective equipment. Some studies have shown nearly identical performance for anterior nares swabs (ANS) and NPS [2]; others have suggested a significantly inferior performance for ANS, particularly later in the course of disease [3]. In addition, some authors have proposed mid-turbinate swabs (MTS) as an alternative to both ANS and NPS. In this paper we set out to answer the question: “When nucleic acid amplification tests RT-PCR tests for SARS-CoV-2 are employed for *initial diagnosis*, what is the relative sensitivity for detection of virus when anterior nares or mid-turbinate samples are used rather than nasopharyngeal swabs?” We focus on *initial diagnosis* to assure that the results of our analysis pertain to the way nucleic acid testing is currently used in SARS-CoV-2 patient management, rather than for treatment monitoring or releasing patients from isolation or quarantine. To answer the question, we conducted a rapid systematic review based on PRISMA principles (a widely accepted set of guidelines for conducting systematic reviews) [4] and compared each sampling method with a composite reference standard [5] We included in our analysis only studies in which NPS and ANS or MT swabs were obtained on the same day, and for which both specimens were analyzed using either a reverse transcription-polymerase chain reaction (RT-PCR) or transcription-mediated amplification (TMA) approach and included in our meta-analysis only studies that demonstrated a low-risk of patient selection bias.

## Methods

**Fig 1:**
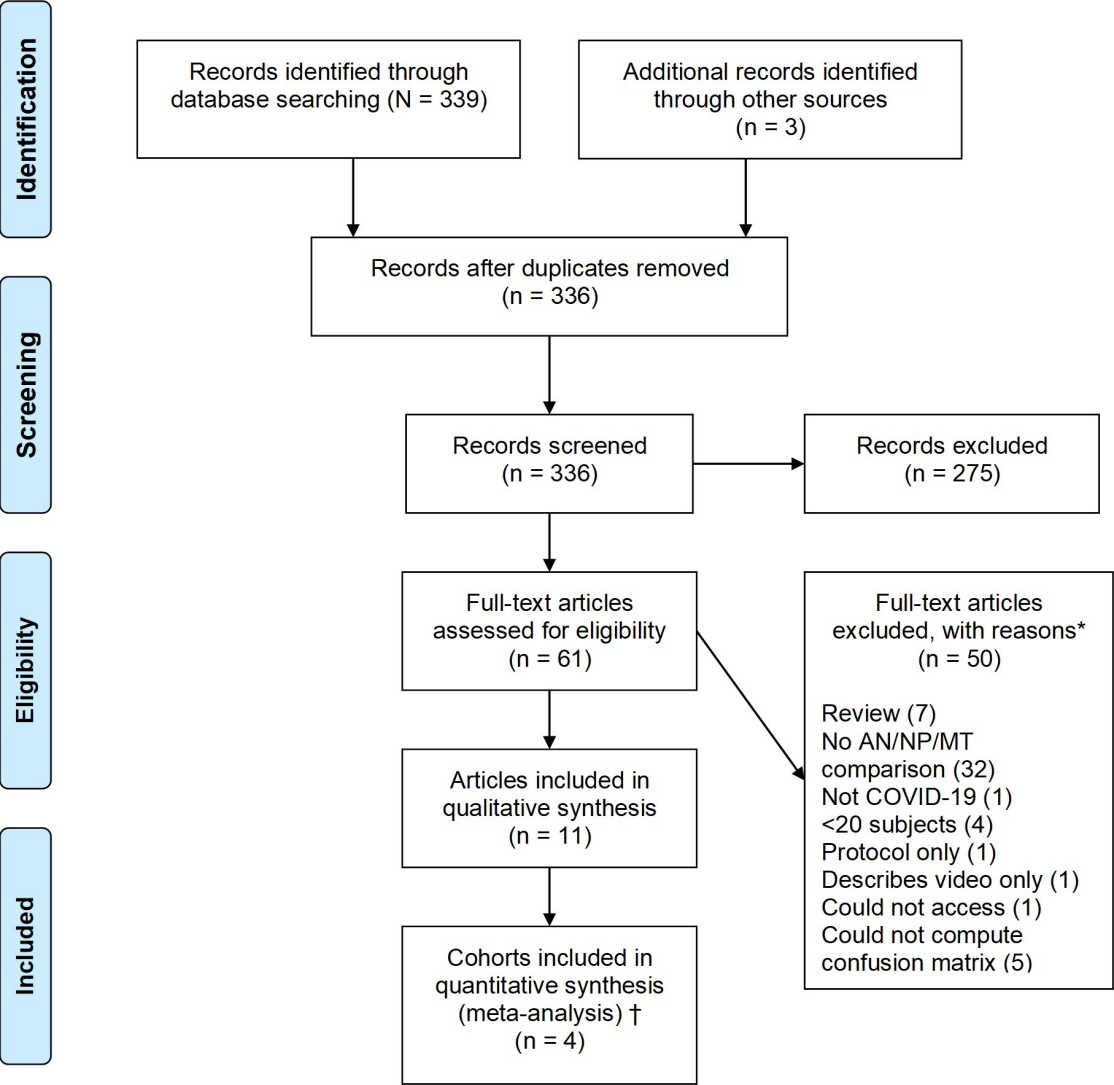
Preferred Reporting Items for Systematic Reviews and Meta-Analyses (PRISMA) flow diagram. *Some papers excluded for more than one reason †Meta-analysis performed with and without studies that had risk of selection bias.

The study is registered on PROSPERO (CRD42020221827). The protocol was not published. We searched PubMed, medRxiv and bioRxiv for studies appearing between January 1, 2020, and March 20, 2021. Preliminary searches showed that the search strategy [nose OR (nasal swab) OR nares) AND (sensitivity OR comparison OR diagnosis) AND (covid19 OR SARS-CoV-2)] was able to capture nearly all relevant papers. In addition, reference lists from review papers and papers identified in the search were also examined for possibly relevant work. Following the initial identification of papers, the titles and abstracts were screened to eliminate papers not meeting the prespecified inclusion criteria. Inclusion criteria required studies with at least 20 specimen pairs (or triads), each consisting of an ANS or MTS and a NPS obtained on the same day. Papers were not excluded based on title and abstract unless they indicated that the paper represented a review, or that they did not compare specimen types based upon testing using either RT-PCR or transcription-mediated amplification (TMA) methods. Papers remaining after this process were rescreened in their entireties. Papers were included only if they presented data in a manner that enabled construction of a confusion matrix of all possible combinations of test results: +/+, -/-, +/-, and -/+. Studies were excluded if they used a different diagnostic device for testing ANS/MTS and NPS. After screening 336 distinct papers, only 11 met inclusion criteria and were analyzed, as shown in the Preferred Reporting Items for Systematic Reviews and Meta-Analyses (PRISMA) flow diagram [6] (Fig 1). No attempt was made to obtain data from the investigators involved in these published studies.

The potential for study-associated bias was evaluated using QUADAS2 [7, 8], which is used to assess patient selection bias and other biases that may be introduced by the manner in which testing is conducted. For example, patient selection bias or spectrum bias (case-mix bias), which occurs when test performance differs among patient populations, was assessed from the perspective of testing as an initial diagnostic method for ambulatory patients because site-specific viral load may change with time. Bias assessment does not constitute a judgement on the quality of the study, which may have been performed to demonstrate assay validity, assessment of recovery, or purposes different than that for which we evaluated potential bias. A predetermined data extraction form included author, study type, inclusion and exclusion criteria, setting, swab and sample types, transport medium, description or manufacturer of nucleic acid amplification assays, and space to record confusion matrices. Evaluations were performed by both authors, who resolved several minor differences regarding the characterization of potential bias after rereading and discussion. Meta-analysis calculations were performed by TJO.

Three papers (4 cohorts) with a low risk of patient selection bias were deemed appropriate to include in a meta-analysis based upon ANS and NPS [2, 9, 10] and these studies were analyzed using a diagnostic effects model (der Simion–Laird) [11] as implemented by OpenMetaAnalyst software program [12]. In order to meet the continuity condition for the calculation, 0.5 was added to all cells.

Because choice of any particular sample type as a “gold standard” yields a biased estimate of relative sensitivity compared to all other sample types, a composite reference standard (CRS) [5] was computed for each study. Neither equivocal results nor assay failures were included in sensitivity calculations, nor in construction of the CRS. Confidence limits for sensitivity (reported in Table 1) were computed using Newcombe’s efficient score method, as implemented in the Vassarstats Clinical Calculator 1 (http://vassarstats.net/). Predictive value calculations were carried out using the MedCalc diagnostic test evaluation calculator (https://www.medcalc.org/calc/diagnostic_test.php). Pre-specified criteria for inclusion in the formal meta-analysis were studies with low-risk of spectrum bias, similar sample types (ANS, MTS or NPS), and similar clinical environments (drive-through / community health center or hospital). Several exploratory (not pre-specified) meta-analyses were also performed.

**Table 1:** Study characteristics and relative sensitivity of anterior nares, nasopharynx, and mid-turbinate sites.

Study	Study Site	Patient Characteristics	Risk of Spectrum Bias	Total Number of Subjects	Percent positive	ANS Sensitivity	NPS Sensitivity	MTS Sensitivity	Self or health care worker	Platform (LOD)
Federman [20]	Hospital and drive-through testing site	Ambulatory and hospitalized. Symptomatic or high exposure risk	High	81	25		19/20	18/20	Healthcare workers	Simplexa 1 COVID-19 Direct Kit 6000 NDU/mL)
							95% (73–100%)	90% (67–98%)		
Berenger [15]	Participant’s homes	Previously tested positive	High	36	81	24/29	27/29		Healthcare workers	LDP
						83% (63–93%)	93% (76–99%)			
Péré [14]	Hospital	Patients suspected for COVID19	Unclear	44	84	33/37	37/37		Healthcare workers	Allplex 2019-nCoV assay
						89% (74–96%)	100% (88–100%)			
Tu [2]	Five ambulatory clinics	Ambulatory subjects with upper respiratory symptoms	Low	498	10	48/51	51/52	50/52	Self	Samples were sent to a reference laboratory for RT-PCR testing; testing specifics were not described
						94% (83–98%)	98% (88–100%)	96% (86–99%)		
Callahan [3]	Drive-through and walk-up testing site	Ambulatory patients with suspected COVID19 or previous positive	High	308	32	47/98	92/98		Healthcare workers	The Abbott Real-Time
										SARS-CoV-2(2700 NDU/mL)
						48% (38–58%)	94% (87–97%)			
Griesemer [9]—Albany	Medical center testing tent	Ambulatory symptomatic and asymptomatic	Low	236	5.2	5/12	12/12		Healthcare workers	LDP
						42% (16–71%)	100% (70–100%)			
Griesemer [9]–New Rochelle	Drive-through testing site	Ambulatory symptomatic and close contacts	Low	227	41	81/93	91/93		Healthcare workers	LDP
						87% (78–92%)	98% (92–100%)			
Kojima [16]	Participant’s homes	Previously tested ambulatory subjects	High	45	64		23/29	23/27*	Healthcare workers	Modified CDC (unmodified CDC is 18000 NDU/mL)
							79% (60–91%)	85% (65–95%)		
McCulloch [13]	Emergency department and drive-through testing center	Ambulatory symptomatic patients, including 27 known-positives	High	185	21		35/38	31/38	Self	LDP
							92% (78–98%)	82% (65–92%)		
Hanson [10]	Drive-through testing center	Symptomatic patients	Low	354	24	70/81	80/81		Self	Hologic Aptima SARS-CoV-2
										TMA test (600 NDU/mL)
						86% (77–93%)	99%			
							(92–100%)			
Pinninti [22]	Hospital	Symptomatic and asymptomatic hospitalized patients	High	40	85		34/34	29/34	Healthcare workers	Modified CDC (200 copies/mL)
							100% (87–100%)	85% (68–94%)		
Liu [21]	Hospital	Confirmed or highly suspected SARS-CoV-2 infection	High	48	54	23/26	26/26		Healthcare workers	Sansure Biotechnology (200 copies/mL)
						88% (69–97%)	100% (84–100%)			

*Two MT specimens were deemed as having insufficient material for analysis. Had these specimens been excluded from the computations for NPS, both would have demonstrated the same performance level (23/27).

** Only data from the AM specimen collection is included in this table.

## Results

Three hundred thirty-six papers were considered for inclusion; of these, 11 studies comprising 12 cohorts met inclusion criteria (Fig 1) [2, 3, 9, 10, 13–17]. A brief summary of the studies is included in this review (Table 1), and more detailed notes about each paper (including the results used in this review) are available in the S1 File. The included cohorts ranged from 38 to 498 patients, with six having fewer than 100 patients. One study separately presented data for two cohorts [9]. The risk of spectrum bias associated with the study population, or method of recruitment, was rated as “high” for seven studies that included patients who had previously tested positive [3, 13, 15–17]. One of these studies was also deemed to have a high risk for “flow and timing” bias [13]. No studies were believed to have a risk of index test bias or reference test bias, in part because the inclusion criteria minimized the opportunity for such bias. The prevalence of SARS-CoV-2 infection within the cohorts, as measured using the CRS, varied from 5.2% to 85%.

Estimates of NPS sensitivity ranged from 79% to 100%, with 7 of the 12 cohorts giving estimates of 98% or greater. Only one study, in which an estimate of 79% for NPS sensitivity was identified [16], estimated NPS sensitivity at less than 90%. Several factors may have contributed to this low estimate. This study included subjects that had previously tested positive for SARS-CoV-2. As a result of study design, positive patients were probably in the later stages of infection. In addition to ANS and NPS, two different saliva samples were collected for these patients and used in computation of the CRS. Previous studies have suggested that saliva is more sensitive than NPS for SARS-CoV-2 detection in the latter stages of disease [18, 19], and exclusion of saliva from the CRS results in an estimate of 100% for NPS sensitivity. This effect is not seen in a study in which testing that included saliva specimens was conducted in a walk-up or drive-through setting that focused on initial diagnosis.

Eight studies examined the sensitivity of anterior nares specimens, arriving at sensitivity estimates ranging from 42 to 94%; five of these studies showed ANS sensitivity between 83 and 89%. In every study and cohort included in our analysis, ANS were inferior to NPS when compared to the composite reference standard, with a relative inferiority ranging from 4% to 46%. MTs was inferior to NPS in five of the six cohorts examined, and appears to be comparable to or greater than that of ANS, varying between 82% and 96% sensitivity [2, 13, 20–22]. Confidence intervals for sample types overlapped significantly for all studies included in this review.

Two papers (Callahan and the Griesemer Albany cohorts) included in this review showed dramatically lower sensitivity for ANS than for NPS [3, 9]. One of these studies (Callahan) found that the reduced sensitivity was independent of the swab type or transport approach [3]. The authors suggested that the sensitivity of ANS is dependent on the sensitivity of the RT-PCR assay employed, and that SARS-CoV-2 patients with lower viral loads may thus get false-negative results when ANS is used in place of NPS. This conclusion was also reached by Pinninti [22]. Given that many of the patients in the Callahan study were tested as part of follow-up after laboratory-confirmed diagnosis, it is possible that more rapid reduction of viral loads in the anterior nares than in the nasopharynx with disease progression accounts for the results of these studies, and that heterogeneity in patient populations may account for differences observed in other studies included in this review. Reduced viral loads in asyptomatic vs symptomatic patients could possibly also account for the reduced sensitivity for ANS in the Albany cohort, since Ct values tend to be higher in asymptomatic than symptomatic patients [23], but viral load information was not published for these patients.

Examination of Table 1 shows no obvious relationship between the prevalence of disease in the tested populations and the sensitivity of ANS, MTS, or NPS identified in those populations, nor does it suggest a relationship between hospitalization and sensitivity. Perhaps because all the testing platforms have low limits of detection, neither was there an obvious relationship between testing platform and relative sensitivity of the swab types. Although both cohorts with poor sensitivity for ANS had these specimens collected by healthcare workers, Table 1 shows that professionally collected specimens provided high sensitivity in most cohorts. Similarly, the study summaries in the supplemental data do not suggest great differences in the performance of different nasal swabbing materials.

**Fig 2:**
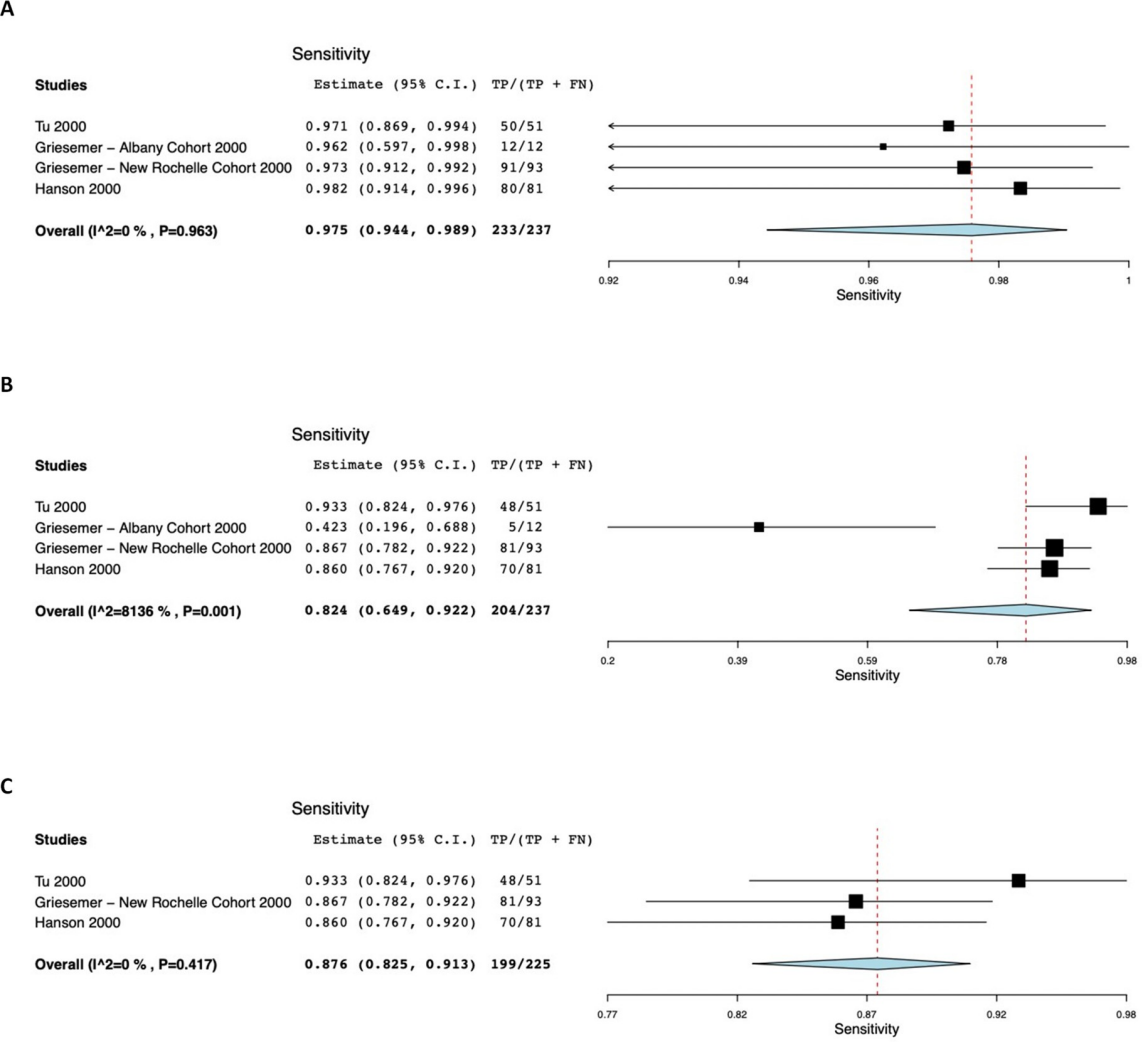
Computer output showing sample sensitivity forest plots and meta-analysis results (studies with low risk of recruitment bias). (A) Results for nasopharyngeal specimens. (B) Results for anterior nares specimens. (C) Exploratory analysis in which the Albany cohort from Griesemer [9] was excluded.

Fig 2 presents computer output for the meta-analyses based on studies of ANS having a low risk of recruitment bias. The pre-specified analysis estimated the sensitivity of NPS, as compared with the composite reference standard, at 98%, with lower and upper bounds of 94% and 99% respectively (Fig 2A). There was no statistical suggestion of heterogeneity (τ^2^ = 0.000, Q[df = 2] = 0.284, *p* = 0.963, I^2^ = 0). In contrast, the sensitivity of ANS (Fig 2B) was estimated at 82% (95% CI 65% - 92%). There was significant heterogeneity (τ^2^ = 0.709, Q[df = 2] = 16.902, *p*<0.001, I^2^ = 81.357). An unplanned exploratory analysis (Fig 1C) that excluded the Griesemer Albany cohort [9] showed AN sensitivity of 88% (95% CI 83% - 91%), with little suggestion of heterogeneity (τ^2^ = 0.000, Q[df = 2] = 1.750, *p* = 0.417, I^2^ = 0). There is no overlap in 95% confidence intervals between ANS and NPS specimens.

**Fig 3:**
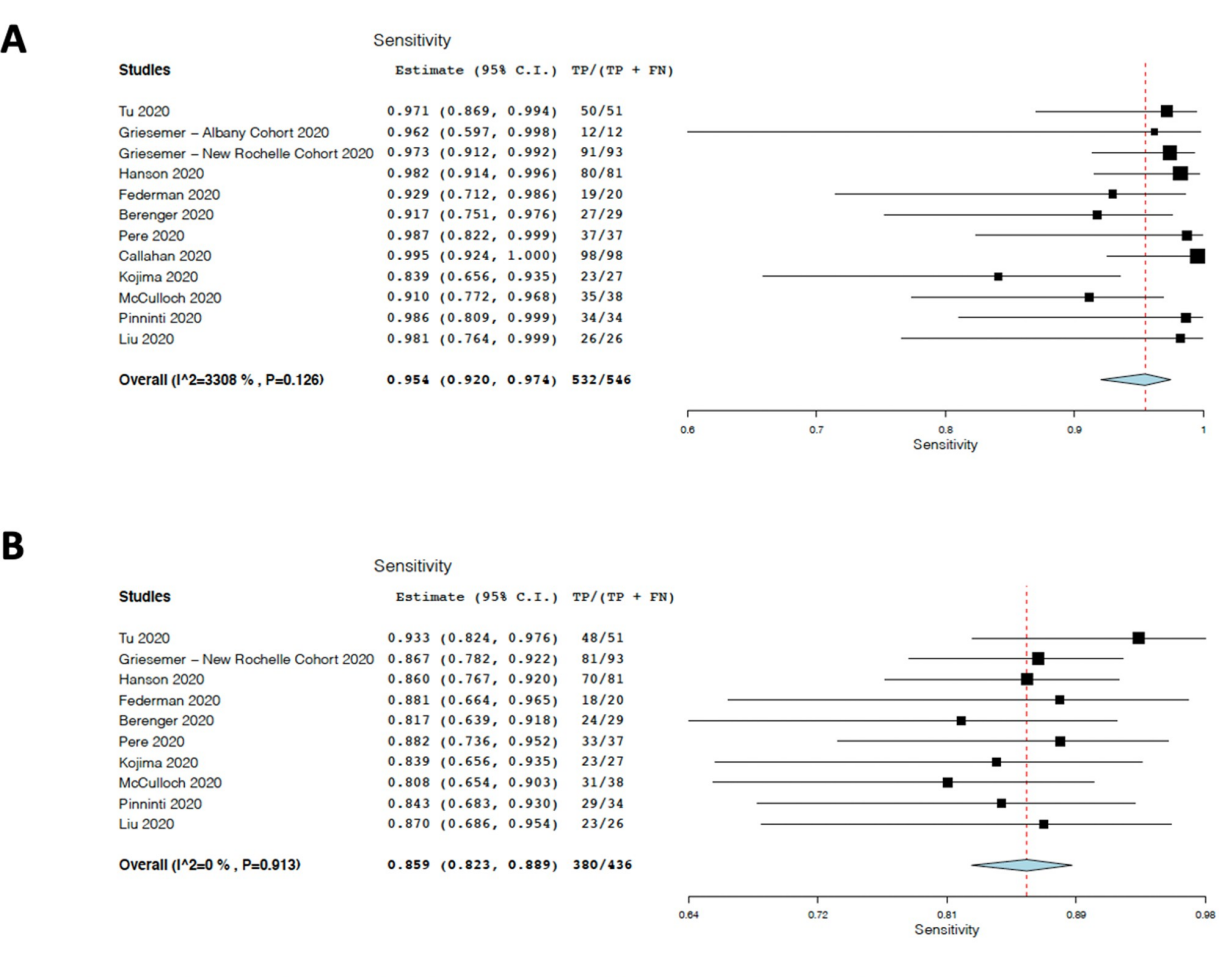
Computer results from unplanned exploratory analysis to include all anterior nasal swab and middle turbinate samples. (A) Results for NPS from all cohorts included in this review. (B) ANS+MTS from all cohorts except the Griesemer [9] Albany cohort and the Callahan [3] cohort. Only ANS results are included from the Tu study [2].

Because visual inspection suggested little difference between the results obtained from studies believed to have a low risk of recruitment bias and studies believed to have a higher risk, as well as little or no difference between ANS and MTS, we conducted a second unplanned exploratory meta-analysis that included all ANS and MTS cohorts. Assessment of NPS showed at most a modest and not statistically significant suggestion of heterogeneity (τ^2^ = 0.337, Q[df = 11] = 16.437, *p* = 0.126, I^2^ = 33.08), and yielded a sensitivity estimate for NPS of 95% (95% CI 92–97%), as seen in Fig 3A. This result is quite similar to that seen when only cohorts with a low risk of recruitment bias were included in the analysis. When the results of ANS and MTS from all studies were combined, there was once again strong evidence for statistical heterogeneity (τ^2^ = 0.882, Q[df = 2] = 70.904, *p*<0.001, I^2^ = 84.486). Removing the Callahan [3] and Griesemer [9] Albany cohorts reduced heterogeneity (τ^2^ = 0.000, Q[df = 11] = 3.969, *p* = 0.913 I^2^ = 0.000) and yielded an estimate of ANS/MTS swab sensitivity of 86% (95% CI 82–89%) (Fig 3B). This point estimate is somewhat higher than that originating from cohorts having low risk of spectrum bias. For this exploratory analysis, there is no overlap between 95% confidence intervals for NPS and ANS/MTS.

## Discussion

Nasopharyngeal swabs have been the “gold standard” for diagnosis of upper respiratory infections, including SARS-CoV-2 infection. The studies we examined have reaffirmed this approach. Only one study included here shows a diagnostic sensitivity less than 90% for NP swabs. Our meta-analysis of cohorts undergoing initial diagnostic testing gives an estimated 98% (95% CI 94% - 99%) sensitivity for NP swabs. These estimates are similar to that obtained by repeat testing [24]. This results in high confidence regarding NP swab sensitivity for initial diagnosis of SARS CoV-2 infection when assessed against the CRS. The meta-analysis suggests noticeably lower sensitivity (95% CI 82% - 88%) for ANS; review of the data in Table 1 suggests than MTS gives results comparable to those of ANS. These results are similar to those found in a recent review and meta-analysis of SARS-CoV-2 testing that used a different methodologic approach [25], and parallel the results seen in influenza testing [26–28]. In a testing environment in which the “true” SARS-CoV-2 infection rate is 10%, the negative predictive value of ANS swabbing, based on the composite reference standard, is estimated as 98.1% - 99.3%, whereas that of NPS is estimated at 99.3% - 99.9%. Although somewhat less sensitive than NPS for detection of SARS-CoV-2, ANS and MTS sample types are more amenable to self-collection, which speeds specimen acquisition and reduces the use of personal protective equipment. This is a significant advantage when testing large numbers of individuals and when more frequent retesting is needed. Since frequent retesting may be more important than test sensitivity for controlling pandemic spread, the reduced sensitivity associated with ANS or MTS may be a reasonable trade-off for throughput. Nevertheless, our analysis strongly suggests that NPS remains the “gold standard” sample for SARS-CoV-2 when used in diagnostic (as opposed to screening) settings in which time and resources are not constrained.

Although variability in nasal swab construction and transport conditions cannot account for the very low sensitivity for ANS seen in two of the studies, it may account for some of the variation seen among the others. Flocked, foam, and spun polyester swabs all function reasonably well, but none is optimal for all operators and transport conditions [29–31]. Nevertheless, the studies meeting inclusion criteria for our systematic review protocol do not provide evidence for the superiority/inferiority of any ANS or MTS swabbing device, transport approach, or for healthcare worker-collected vs self-collected ANS/MTS. Although the data suggest that, least with some assay systems, ANS may be less effective in subjects with low viral loads than it is in those with higher viral loads, the data that we examined does not provide a robust test of ANS efficacy in the identification of asymptomatic infection. Data in the literature strongly suggests that asymptomatic patients have lower viral loads than symptomatic patients, but also show that many asymptomatic patients are effectively diagnosed using ANS [23].

Our analysis is limited by the small number of cohorts which met inclusion criteria; inclusion of studies included fewer than 20 subjects might have provided additional information. The use of an abbreviated search strategy may miss publications that otherwise may have satisfied inclusion criteria. The use of a CRS, which defines the false-positive rate as zero for all assays, may introduce a downward bias in the estimates of sensitivity [32]. This bias varies somewhat based on study size and precise cell frequencies. The computational requirement to add a constant to cells containing zero also creates a downward bias in sensitivity estimates, which is greatest for studies in which the lowest sensitivities are identified. This may have resulted in a downward bias of up to 4% in the meta-analysis estimate of ANS sensitivity. Finally, the studies included in the review demonstrate considerable heterogeneity in assay design, patient population, and disease prevalence. Thus, this systematic review and meta-analysis may provide more insight into the range of possible results than they do for the specific performance in any given setting.

This systematic review also has several strengths. It focuses on sample sets that were taken at the same time, which differs from most other systematic reviews. The number of samples is substantially greater than that of any individual study based on simultaneous sample comparison, and allows sensitivity estimates based on meta-analysis. Furthermore, our meta-analysis focuses on initial diagnosis of infection in patients who were not previously tested positive for SARS-CoV-2 or hospitalized for treatment. This may yield a more accurate assessment of performance in community-based initial testing, and provides evidence that, while ANS is inferior to NPS for initial diagnosis, it nevertheless retains high negative predictive value in most populations. In the absence of a “gold standard” for diagnosis of SARS-CoV-2 infection, the CRS is a relatively unbiased approach to a “reference standard” and is more likely to provide a better sense of the true performance difference associated with various sampling approaches than intrinsically biased comparisons. Our conclusions are similar to those found in a recently published systematic review and meta-analysis which was performed using somewhat different methodology, lending strength to our conclusions [25].

During this pandemic, the medical and scientific community has come together in an unprecedented collaborative manner to offer multiple testing options and publish research to inform laboratory testing decisions. By conducting this systematic review, we are able to provide unbiased evidence to colleagues in medicine and public health, that despite minor differences in diagnostic sensitivity, the ANS and MTS testing may be reasonable alternatives given the practical limitations associated with NPS.

## Supporting information

S1 Checklist PRISMA 2009 checklist. (DOC)

S1 File Supplemental data: Study summaries and confusion matrices. (DOCX)
