# Supplementary material for: Relative sensitivity of anterior nares and nasopharyngeal swabs for initial detection of SARS-CoV-2 in ambulatory patients: Rapid review and meta-analysis
Source: PLoS One. 2021 Jul 20;16(7):e0254559. doi: 10.1371/journal.pone.0254559 (PMC8291630; doi:10.1371/journal.pone.0254559)
Supplement: S1 File — (DOCX) [file pone.0254559.s002.docx]

**S1 File**

**Supplemental Data: Study Summaries and Confusion Matrices**

**Federman**

One hundred eight-one Department of Veterans Affairs patients recruited from a “drive-through outpatient testing center, emergency department, and inpatient wards; a breakdown was not provided. Healthcare providers collected both mid-turbinate (ESwab 480C, Copan, Murrieta, CA) and nasopharyngeal swabs [Becton, Dickinson and Company H192(07) flexible fine-tip flocked nasopharyngeal swab], which were transported in Amies solution and in Universal Viral Transport medium (Becton Dickinson, Franklin Lakes NJ) respectively, prior to testing using the Simplexa 1 COVID-19 Direct Kit (DiaSorin Molecular LLC, Cypress CA).

Data is believed to have a high risk of spectrum bias due to the inclusion of inpatients who had been laboratory diagnoses of SARS-CoV-2 from one day to 24 days prior to this study.

|  | | Mid-Turbinate Swab | |
| --- | --- | --- | --- |
|  |  | Positive | Negative |
| Nasopharyngeal Swab | Positive | 17 | 2 |
|  | Negative | 1 | 161 |

**Berenger**

Investigators obtained anterior nares, oropharynx and nasopharynx specimens from individuals who previously had tested positive for SARS-CoV-2. Nasal swabs were obtained using the APTIMA Unisex Collection Kit (Hologic Inc., San Diego CA) and transported in the included medium. Oropharyngeal swabs were collected with the APTIMA Multitest Collection Kit (Hologic Inc., San Diego CA) and also transported in the enclosed medium. Nasopharyngeal swabs were collected using the Flexible Mini Tip Flocked Swab (Copan S.P.A, Italy) and transported in Universal Transport Media (UTM, Copan S.P.A. Italy). All specimens were tested using a laboratory-developed procedure targeting E and RdRp genes at the Alberta Public Health Laboratory.

The study data is considered to have a high risk of spectrum bias for initial diagnosis, since subjects had all been previously diagnosed as positive prior to swabbing for this study.

|  | | Anterior Nares Swab | |
| --- | --- | --- | --- |
|  |  | Positive | Negative |
| Nasopharyngeal Swab | Positive | 22 | 5 |
|  | Negative | 2 | 7 |

**Péré**

Forty-four patients ranging from 18 to 94 years were prospectively enrolled in a study in which they provided a AN and an NP sample from a single nare. The NP swab was transported in virus transport medium (VTM) (Xpert viral transport medium; Cepheid), and the AN swab was transported in 0.9% saline. Testing was performed using the Allplex 2019-nCoV assay (Seegene, Seoul, Korea).

Risk of spectrum bias in the study data is rated as unclear, because the paper does not clearly state whether the patients were presenting for initial diagnosis with a spectrum of disease, or whether they had been hospitalized.

|  | | Anterior Nares Swab | |
| --- | --- | --- | --- |
|  |  | Positive | Negative |
| Nasopharyngeal Swab | Positive | 33 | 4 |
|  | Negative | 0 | 7 |

**Tu**

Investigators recruited 530 patients with symptoms of covid19 from five ambulatory clinics, and obtained NP swabs (Puritan 25-800-2PDBG), as well as self-collected tongue swabs (FLOQSwab 502CS01, Copan, Murrieta, CA), nasal swabs (Puritan 25-1506 1PF100, Puritan Medical Products, Guilford, ME), and mid-turbinate swabs (MDL NasoSwab A362CS02.MDL, 56380CS01 for adults and 56780CS01 for pediatrics, Medical Diagnostic Laboratories LLC, Hamilton NJ). Samples were sent to a reference laboratory for RT-PCR testing; testing specifics were not described. Only the confusion matrices for NP, anterior nares and mid-turbinate swabs are presented below, and composite reference standards were constructed for each 2 x 2 table.

Data are believed to demonstrate a low risk of spectrum bias, due to inclusive prospective enrollment.

|  | | Anterior Nares Swab | |
| --- | --- | --- | --- |
|  |  | Positive | Negative |
| Nasopharyngeal Swab | Positive | 47 | 3 |
|  | Negative | 1 | 447 |

|  | | Mid-Turbinate Swab | |
| --- | --- | --- | --- |
|  |  | Positive | Negative |
| Nasopharyngeal Swab | Positive | 50 | 2 |
|  | Negative | 0 | 452 |

**Callahan**

Investigators recruited 308 individuals tested for SARS-CoV-2, based on clinically suspected COVID-19 infection or follow up after previous SARS-CoV-2-positive RT-PCR testing; the number of subjects who had previously tested positive and were therefore recovering from disease was not reported. Subjects first provided a healthcare provider-performed anterior nares specimen, then a standard NP swab. Two swab collection protocols and three sets of transport conditions were employed. The Abbott Real-Time SARS-CoV-2 (Abbott Diagnostics, Des Plaines, IL) assay was employed for all specimens.

Investigators found high concordance only for subjects demonstrating more than 1000 copies/ML. The confusion matrix below shows the results for all subjects tested, regardless of collection method or transport medium, since the differences among groups were modest and probably not significant.

Data is considered to have a high risk of spectrum bias due to the inclusion of subjects who had previously been tested and were recovering from disease at the time of study.

|  | | Anterior Nares Sample | |
| --- | --- | --- | --- |
|  |  | Positive | Negative |
| Nasopharyngeal Swab | Positive | 47 | 51 |
|  | Negative | 0 | 210 |

**Griesemer**

Two hundred thirty-six asymptomatic and symptomatic subjects were enrolled, without exclusion, from a testing tent operated by a medical center. An additional group of 227 symptomatic subjects and close contacts was enrolled from a drive-through testing site where the COVID19 positivity rate was known to be high due to a nearby super spreader event. Subjects each provided a nasal and a NP swab which were transported in Molecular Transport Medium (Longhorn Vaccines and Diagnostics, LLC., San Antonio TX). They also gave a saliva sample which was taken neat, held cold, and transported within 24 hours to the laboratory. RTPCR testing followed RNA extraction and was performed within 72 hours after specimen collection.

Neither cohort was recruited in a manner that suggests more than a low risk of spectrum bias. The spectrum of disease might be different between the two sites.

Albany Medical Center screening tent

|  | | Anterior Nares Sample | |
| --- | --- | --- | --- |
|  |  | Positive | Negative |
| Nasopharyngeal Swab | Positive | 5 | 7 |
|  | Negative | 0 | 224 |

New Rochelle drive-through testing center*

|  | | Anterior Nares Sample | |
| --- | --- | --- | --- |
|  |  | Positive | Negative |
| Nasopharyngeal Swab | Positive | 81 | 10 |
|  | Negative | 0 | 134 |

*Two additional positives, missed by both NP and AN swab, were found by saliva testing.

**Kojima**

This study recruited ambulatory patients, including symptomatic adults who had been previously tested at a drive-through center, first responders, and possibly exposed law enforcement personnel. A specific attempt was made to recruit 30 subjects who were negative for SARS-CoV-2 virus and 30 subjects who were positives. Investigators obtained saliva, clinician-supervised self-collected mid-turbinate (CLASSIQSwabs™, Copan Diagnostics, Murrieta, CA, USA) swabs and clinician-collected posterior nasopharyngeal (CLASSIQSwabs™, Copan Diagnostics, Murrieta, CA, USA) swabs during visits to subject homes. The timing of the second specimen acquisition with respect to initial testing is unclear. Specimens appear to have been transported to the laboratory in DNA/RNA Shield™ solution, (Zymo Research Corp., Irvine, CA, USA), where RNA was extracted and and testing using a modified CDC assay.

This study was assessed as having a high risk of spectrum bias due to a specific attempt to balance the numbers of patients testing positive and negative for SARS-CoV-2 and inclusion of individuals previously found to be positive.

|  | | Mid-Turbinate Sample | |
| --- | --- | --- | --- |
|  |  | Positive | Negative |
| Nasopharyngeal Swab | Positive | 19 | 4 |
|  | Negative | 4 | 16 |

Subjects for which MT swabs were deemed “quantity not sufficient” are not included in this table, and were not considered in computation of the composite reference standard. Several specimen types which are not reported in this table were, however, used in computing the composite reference standard.

**McCulloch**

Investigators recruited 185 subjects from ambulatory symptomatic outpatients who tested positive for SARS-CoV-2 using a clinician-collected NP swab SARS-CoV-2 and from symptomatic healthcare workers presenting to drive-through SARS-CoV-2 testing clinics. Members of the first group were contacted by members of the research team following notification of a positive result, and after consent, had a home swab kit including a mid-turbinate swab (Copan FloqSwab 56380CS01, Copan Diagnostics, Murrieta, CA) and Universal Transport Medium (UTM) (Becton Dickinson, Franklin, NJ) delivered to their home within hours. The second group received a home swab kit at the time of testing while making their drive-through clinic appointment. The patient population included both individuals who were being tested for the first time, and individuals who were being tested for “test of cure.”

The study is considered to have a moderate risk of spectrum bias due to the inclusion of subjects who had previously tested positive for SARS-CoV-2, together with the simultaneous exclusion of patients who had not tested positive. However, since the positive tests were taken from a randomly sampled population, they probably do not represent a biased spectrum of positive patients (for diagnostic sensitivity calculations) disease in an initially diagnosed population. There is a high risk of “flow and timing” bias due to the differences in the intervals between specimen acquisition and testing for NPS and MTS samples.

|  | | Mid-Turbinate Sample | |
| --- | --- | --- | --- |
|  |  | Positive | Negative |
| Nasopharyngeal Swab | Positive | 28 | 7 |
|  | Negative | 3 | 140 |

**Hanson**

This study included 354 symptomatic adults from a drive-through testing center. Subjects provided saliva samples and swabbed both nostrils prior to NPS sampling. It is unclear whether an RNA extraction step was performed. Although data for this study included nasal specimens, saliva specimens and NPS, the data was not presented in a manner that allows computation of a composite reference that includes all three sample types. For this reason, the sensitivity presented in Table 1 for ANS and NPS is based upon a composite reference based only on those two sample types.

The data may reflect flow and timing bias, since the order in which specimens were obtained was not randomized, but this bias, if present would favor anterior nares samples. We believe the risk to remain low, despite this failure to randomize. We believe the study to have a low risk of patient selection bias.

|  | | Anterior Nares Sample | |
| --- | --- | --- | --- |
|  |  | Positive | Negative |
| Nasopharyngeal Swab | Positive | 69 | 11 |
|  | Negative | 1 | 273 |

**Pinninti**

Forty hospitalized patients with confirmed covid19 infections were enrolled. Paired MT and NP specimens were collected weekly by the same medical provider from both nares, placed in an unspecified transport medium and stored at -80C prior to RNA extraction (QAmp viral RNA mini-kit, Quiagen, Valencia, CA) and RT-PCR using a modified CDC assay with a detection limit of between 100 and 200 copies/mL. The study is considered to have a high risk of patient selection bias due to the inclusion of known positive, hospitalized patients. The data may reflect flow and timing bias, since the order in which specimens were obtained was not randomized. Data from only the first set of paired specimens is included in the confusion matrix below.

|  | | Mid-Turbinate Sample | |
| --- | --- | --- | --- |
|  |  | Positive | Negative |
| Nasopharyngeal Swab | Positive | 29 | 5 |
|  | Negative | 0 | 6 |

**Liu**

The study included 48 confirmed or highly suspected, hospitalized, covid19 patients who provided simultaneous nasopharyngeal, nasal, oropharyngeal and anal swabs in both the morning and the afternoon of the same day. No information is provided on whether the order of specimen acquisition was randomized. Details of swab construction were not provided, nor was information on specimen transport. Assay was performed by an RT-PCR method (Sansure Biotechnology Ltd, China) following RNA extraction. The study is believed to have a high risk of patient selection bias as a result of including only hospitalized patients. The data may reflect flow and timing bias, since the order in which specimens were obtained was not randomized.

Morning Sample

|  | | Anterior Nares Sample | |
| --- | --- | --- | --- |
|  |  | Positive | Negative |
| Nasopharyngeal Swab | Positive | 23 | 3 |
|  | Negative | 0 | 22 |

Afternoon Sample

|  | | Anterior Nares Sample | |
| --- | --- | --- | --- |
|  |  | Positive | Negative |
| Nasopharyngeal Swab | Positive | 13 | 4 |
|  | Negative | 1 | 30 |
